# Secondary Metabolites and Biological Activities From *Achillea gypsicola* Hub‐Mor. Under Foliar Applications of Chitosan and Salicylic Acid

**DOI:** 10.1002/fsn3.70225

**Published:** 2025-05-12

**Authors:** Muhammed Akif Açıkgöz, Ebru Batı Ay, Beril Kocaman

**Affiliations:** ^1^ Department of Field Crops Faculty of Agriculture, Ordu University Ordu Turkey; ^2^ Department of Plant and Animal Production Suluova Vocational School, Amasya University Amasya Turkey

**Keywords:** Asteraceae, bioactive compounds, growth stages, minimum inhibitory concentration

## Abstract

Elicitors play a crucial role in plant defense systems, often leading to an enhancement in secondary compounds across various species. The aim of this paper was to assess the effect of elicitors of salicylic acid (SA) and chitosan (CTS) applied to the leaves on essential oil (EO) composition of *Achillea gypsicola* and some terpenoids and biological activity. Treatments with SA at doses of 0, 0.5, 2, and 8 mM, and CTS at 0, 2, 4, and 8 g L^−1^ were evaluated. Two harvests were conducted between 2021 and 2022. Elicitors SA at 8 mM and CTS at 2 g L^−1^ led to an increase in EO productivity. The application of elicitors, particularly at doses of 2 mM SA and 4 g L^−1^ CTS in *A. gypsicola*, has the potential to stimulate camphor production. Additionally, TPC and TFC were the highest in treatments of SA 2 mM and CTS 8 g L^−1^. LC–MS/MS analysis indicated that the synthesis of camphor and 1,8‐cineole enhanced significantly compared to the control group, with enhancements of 213.2% at 8 g L^−1^ CTS and 125.1% at 4 g L^−1^ CTS, respectively. The EOs are especially effective against *Escherichia coli* , *Pseudomonas aeruginosa* , *Staphylococcus aureus* , and *Listeria monocytogenes* .

## Graphical abstract

Foliar practice of elicitors enhanced yarrow bioactive compounds (BCs). Full flowering stage is effective in the synthesis of BCs. Camphor and 1,8‐cineole were increased by 2.13 and 1.25 fold, respectively.

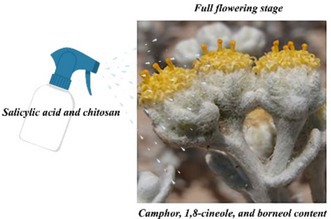

## 1 Introduction

The genus *Achillea* includes 115 species globally, with 59 species found in Turkey, of which 31 are endemic (Açıkgöz 2020). This genus comprises perennial plants that are known for their fragrant flowers and are widely distributed in Europe, the Middle East, and Anatolia. Phytochemical analysis of these plants plays a crucial role in pharmaceutical and medicinal preliminary studies (Açıkgöz et al. 2022, 2023). Up until now, studies have demonstrated that species within the *Achillea* genus possess antimicrobial, antioxidant, antidiabetic, and anti‐inflammatory effects (Farajpour et al. 2024; Huang et al. 2024; Kbaydet et al. 2024). It was exposed that the main compounds of essential oils (EOs) of this genus are bioactive compounds such as borneol, 1,8 cineole, and santolina alcohol (Vojoudi et al. 2024) α‐bisabolol (Ilardi et al. 2024) camphor, nerolidol, and piperitone (Niazipoor et al. 2024) α‐terpineol, caryophyllene, and chamazulene (Yapar et al. 2024) with a significant correlation of these compounds among *Achillea* species in earlier research. As noted by Amssayef et al. (2024), Gabbanini et al. (2024), Ilardi et al. (2024), and Raudone et al. (2024), while extensive research has been conducted on the composition and biological processes of EOs in plants, the primary focus has been on EO content due to its economic significance. The health advantages of medicinal and aromatic plants (MAPs) are not limited to their EOs; they also encompass phenolic compounds (Najafian et al. 2022; Kemal et al. 2023). Despite numerous studies on plants, there is a lack of information on enhancing phenolic compound levels and improving the antioxidant capacity of *A. gypsicola* through cultural practices, with most research limited to in vitro culture systems. Phenolic compounds, including phenolic acids and flavonoids, are secondary metabolites (SMs) exhibited in the non‐volatile parts of plants. These metabolites are known for their diverse biological activities, such as antioxidant and antimicrobial properties (Betlej et al. 2023), anti‐inflammatory (Ugbogu et al. 2024), and anticancer (Ay et al. 2023a). They also inhibit enzymes like pancreatic lipase, α‐Glucosidase, and α‐Amylase (Tundis et al. 2023). Due to these useful characteristics, phenolic compounds are extensively utilized in the cosmetic industries (Ben Hsouna et al. 2023), pharmaceuticals (Gharsallah et al. 2023), and food (Gholamipourfard et al. 2021). The levels and types of these bioactive compounds in medicinal and aromatic plants (MAPs) can vary based on factors such as the plant's growth stage (Açıkgöz 2019), genetic makeup, climatic conditions (Sharma et al. 2019), environmental ingredients (Pant et al. 2021), extraction techniques (Zengin et al. 2023), cultivation methods, and plant parts used (Ay et al. 2023a, 2023b). Thus, applying practices that consider these factors can enhance the synthesis of SMs to the wanted levels. For instance, understanding which metabolites are synthesized inclusively at specific periods and promoting plants with appropriate nutrients can enhance the efficiency of these metabolites (Dilek et al. 2022; Kırgeç et al. 2023). Among these bio‐stimulants, chitosan (CTS) and salicylic acid (SA) have garnered significant attention recently. Natural polymers, such as CTS, have extensive applications in the food industry and agriculture due to their renewable nature, biodegradability, biocompatibility, and low cost (Zaini et al. 2024). CTS, derived from the deacetylation of chitin found in the exoskeletons of crabs, shrimps, and crustaceans, is the second most used polymer after cellulose (Nath et al. 2024). Its high solubility in water and biocompatibility (Mirbagheri et al. 2024) have led to its increased use as a stimulant and bio‐stimulant in plants (Rani et al. 2023; Shrestha et al. 2023; Stasińska‐Jakubas et al. 2024), making it popular in organic farming practices within sustainable agricultural systems (Dhiman et al. 2023; Riseh et al. 2023; Sun et al. 2023; Medeiros et al. 2024). SA is vital for plant defense against pathogens and can be produced through either the phenylalanine or isochorismate pathways (Açıkgöz 2019). When used externally, SA elicits responses akin to those activated by pathogen attacks or other external factors (Mishra et al. 2024; Zou et al. 2024). This hormone triggers signaling pathways that activate the recording of different genes, leading to the buildup of defense‐related compounds like alkaloids, terpenoids, and polyphenols (Li et al. 2024).

*A. gypsicola*, listed as vulnerable in the Data Book, is an endemic species of Central Anatolia in Turkey. This plant possesses antioxidant and antimicrobial properties and consists of camphor, 1,8‐cineole, borneol, menthone, and menthol, which are used for various purposes in the cosmetics and pharmaceutical industries. Until now, the investigations have exposed features of this species, such as the SM accumulation in cell suspension culture (Açıkgöz 2019), antimicrobial activities, and chemical composition of both extractions and EO of aerial parts (Açıkgöz 2019, 2020). To date, there have been no studies aimed at enhancing the phytochemical composition and biological properties of *A. gypsicola*.

It seems that the information on *A. gypsicola* is quite limited. The number of these few products was determined by collecting the secondary metabolites of this species from nature and determining them qualitatively and quantitatively. Considering the limited information about *A. gypsicola* cultivation, this study is important to determine the effect of cultural practices on the species. In addition, enhancing certain secondary metabolites through cultural practices is crucial for their pharmacological benefits. Thus, this study aims to (1) assess the impact on SA and CTS essential oil components, (2) investigate the variations in total phenolic and flavonoid content, (3) identify the changes in borneol, camphor, and 1,8‐cineole levels, and (4) evaluate the antioxidant capacity and antimicrobial activities.

## 2 Materials and Methods

### 2.1 Field Description and Experimental Design

Yarrow (*Achillea gypsicola* Hub. Mor.) seeds were sourced from the Department of Field Crops at Ordu University. The species was identified by Assoc. Prof. Dr. Sevda Türkiş from Ordu University. The voucher specimen, numbered 5810, is housed in the Herbarium of the Department of Biology at Gazi University, Ankara, Turkey. Seeds were collected on August 11, 2020, then cleaned and stored in vacuum‐sealed cloth bags at +4°C. The field experiment commenced in 2020 in Corum–Iskilip, Turkey (latitude 40°44′ N, longitude 34°28′ E). Seeds were planted at a depth of 3–4 cm. The soil had a light loamy texture with a pH of 8.78, an electrical conductivity (EC) of 0.447 dS m^−1^, organic matter content of 0.12%, available potassium (K) and phosphorus (P) levels of 72 and 1.7 mg kg^−1^, respectively, and a total nitrogen (N) content of 0.015%. SA was applied to plants at three concentrations: 0 (water 90% + ethanol 10%), 0.5, 2, and 8‐mM solutions (w/v %). Similarly, CTS was applied at three different concentrations: 0 (water 90% + ethanol 10%), 2, 4, and 8 g L^−1^. Stock solutions for both treatments were prepared and applied separately via foliar spraying on the full flowering period (June 9, 2021, and June 13, 2022). Climatic characteristics of the experimental site are detailed in Table 1. Harvesting occurred on August 22, 2022, with 15 plants sampled. The plants were dried at 22°C–24°C in a well‐ventilated shaded area and then ground into a fine powder using a mixer. The research followed a completely randomized block plan with four replications.

**TABLE 1:** Results of the climatic characteristics of the experimental site.

Mounts	Monthly average temperature (°C)			Monthly total rainfall (mm)			Monthly average relative humidity (%)		
	2020	2021	2022	2020	2021	2022	2020	2021	2022
January	−0.3	0.9	0.8	39.1	38.6	38.5	75.0	71.6	73.0
February	1.2	2.7	0.8	29.3	36.6	36.2	69.9	64.5	71.3
March	5.0	6.7	7.3	39.0	46.9	45.0	65.5	65.3	69.8
April	10.5	11.5	10.4	46.4	44.5	45.5	62.4	62.1	60.9
May	15.0	16.5	15.1	61.7	51.0	55.0	62.2	55.3	60.3
June	18.5	20.6	19.5	55.0	40.2	53.0	59.3	57.1	59.8
July	21.3	24.2	24.1	19.9	14.8	16.3	53.3	52.6	50.3
August	21.4	24.3	22.3	15.1	14.6	13.9	52.5	51.3	53.9
September	17.4	19.6	20.1	21.7	17.9	18.4	54.6	53.6	52.8
October	12.2	13.9	12.9	27.1	33.4	29.7	60.8	52.8	59.9
November	6.4	7.3	5.8	33.0	31.9	32.3	67.3	68.1	65.8
December	1.9	2.8	3.0	43.6	43.2	43.1	73.9	72.1	75.0

### 2.2 Chemicals

The following chemicals were sourced from Sigma‐Aldrich (St. Louis, MO): Thiobarbituric acid TBA – Sigma T5500, ethanol Sigma 32221, CAS No: 64‐17‐5, Trolox Sigma‐Aldrich 238813, CAS No: 53188‐07‐1, aluminum chloride AlCl_3_, chitosan (C_6_H_11_O_4_N)n Sigma, CAS No: 9012‐76‐4, dipotassium hydrogen orthophosphate K_2_HPO_4_ Sigma P5504, CAS No: 16788‐57‐1, potassium dihydrogen phosphate KH_2_PO_4_ – Sigma P5655, CAS No: 7778‐77‐0, guaiacol Sigma G5502, CAS No: 90‐05‐1, glacial acetic acid Sigma 27225, hydrogen peroxide H_2_O_2_ – Sigma 18304, CAS No: 7722‐84‐1, and potassium iodide KI – Sigma 793582, CAS No: 7681‐11‐0. Additionally, the following reagents were obtained from Merck (Darmstadt, Germany): Salicylic acid HOC_6_H_4_COOH – Merck 818731, CAS No: 69‐72‐7, Sodium hydroxide NaOH – Merck 106462, CAS No: 1310‐73‐2, gallic acid Merck CAS No: 149‐91‐7, quercetin Merck CAS No: 849061‐97‐8 and sodium carbonate Na_2_CO_3_ – Merck 106395, CAS No: 497‐19‐8.

### 2.3 Extract Preparation From Plant Material

The samples were first rinsed with tap water to remove soil residues and then left to air‐dry on drying paper in a controlled environment for 2 weeks. Once dried, the marked samples were stored in jars. From each sample, 5 g were correctly assessed and placed into boxes. Each jar was then filled with 200 mL of methanol, and the mixtures were macerated in an ultrasonic bath for 3 h. The solutions were filtered to divide the liquid, and the rest was kept. Finally, the methanol evaporated.

### 2.4 Secondary Metabolites Analysis

#### 2.4.1 Determination of Total Phenolic Content (TPC)

The TPC of methanol extracts was determined using the Folin–Ciocalteu reagent and gallic acid (GA) standard from Sigma‐Aldrich Co (St. Louis, MO, USA), based on the method by Siddhuraju and Manian (2007) with slight modifications. In a volumetric flask, 1 mL of the sample extract was mixed with 0.3 mL of a saturated sodium carbonate (Na_2_CO_3_) solution and 0.1 mL of Folin–Ciocalteu reagent. The volume was then adjusted with double‐distilled water, and the solution was incubated at room temperature in the dark for 1 h. The TPC was measured at 765 nm using a UV–visible spectrophotometer, with a calibration curve created using GA as the standard, and the results were shown as mg gallic acid equivalents (GAE) per g of dry weight. Each test was performed in triplicate.

#### 2.4.2 Determination of Total Flavonoid Content (TFC)

The TFC was detected using a modified version of the method described by Zhishen et al. (1999). In brief, 1 mL of the plant extract was mixed with 2 mL of double‐distilled water in a tube. Subsequently, 0.15 mL of 0.5 M NaNO_2_ and 0.15 mL of 0.3 M AlCl_3_ were added. After a 5‐min interval, 1 mL of NaOH was introduced to the mixture. The solution was then developed for 30 min, and the TFC was rated at 510 nm using a UV–visible spectrophotometer (Thermo Fisher, Model G10S‐UV–Vis, USA). The TFC was quantified based on a quercetin standard calibration curve; the values were shown as mg quercetin equivalents (QE) per g of dry weight. All tests were performed in triplicate.

#### 2.4.3 Antioxidant Activity

##### 2.4.3.1 *DPPH* (2,2‐Diphenyl‐1‐Picrylhydrazyl) Radical Scavenging Assay

To evaluate the suspension's effectiveness in neutralizing the DPPH radical, 0.1 mL of the suspension was mixed with 1 mL of DPPH solution and 4 mL of methanol. The mixture was kept in the dark at room temperature for 30 min, following the method described by Brand‐Williams et al. (1995). Absorbance was determined at 517 nm using a UV–visible spectrophotometer. The scavenging activity was recorded using the formula (%) = [1 – (A 517 nm, sample/A 517 nm, control)] × 100. All tests were performed in triplicate.

##### 2.4.3.2 Ferrous Ions Chelating Assay

The ability to chelate ferrous ions was evaluated using a method adapted from Decker and Welch (1990). In this test, 1 mL of the sample or source solution (ranking from 25 to 400 μg mL^−1^) was mixed with 1 mL of acetate buffer (0.1 M, pH 4.9) and 0.1 mL of FeCl_2_ (2 mM). Subsequently, 0.2 mL of ferrozine (5 mM) was added. The reaction was allowed to proceed for 30 min at room temperature (25°C), resulting in a purple‐colored complex with an absorption peak at 562 nm. The chelating activity was calculated using the formula (%) = [1 – (A 562 nm, sample/A 562 nm, control)] × 100. All tests were performed in triplicate.

#### 2.4.4 AntiMicrobial Screening

##### 2.4.4.1 Microorganisms Tested

The antimicrobial properties of EOs were assessed against a variety of 10 microorganisms. The antibacterial effects were examined on three Gram‐negative bacteria: *Proteus vulgaris* ATCC 13315, *Pseudomonas aeruginosa* ATCC 9027, and *Escherichia coli* ATCC 25922, as well as four Gram‐positive bacteria: *Listeria monocytogenes* ATCC 7644, *Staphylococcus aureus* ATCC 6538, *Bacillus subtilis* ATCC 6059, and *Streptococcus faecalis* ATCC 8043. Additionally, the anti‐fungal activity was tested on three fungal species: *Aspergillus niger* ATCC 16404, *Saccharomyces cerevisiae* (ATCC 9763), and *Candida albicans* ATCC 10231.

##### 2.4.4.2 Disc Diffusion Method

The antimicrobial properties of *A. gypsicola* EOs were evaluated using the disc diffusion method, following the procedure detailed by Sacchetti et al. (2005). Initially, parent cultures were fixed by inoculating Mueller Hinton Agar plates with microorganisms and incubating them at 37°C for 24 h. Fungal cultures were grown on Sabouraud Dextrose Agar at 25°C for 48 h. Bacterial cultures were cultivated in Mueller Hinton Broth, while fungal cultures were grown in Sabouraud Dextrose Broth, followed by incubation. Petri dishes were inoculated with mother cultures using appropriate sterile media to achieve a microorganism concentration of 10^8^ colony‐forming units (CFU) per mL. In the disc diffusion test, 6 mm diameter sterile filter paper discs were saturated with 50 μL of EO and placed on the inoculated agar plates. Standard antibiotic discs (nystatin, gentamicin, ampicillin, and penicillin) served as positive controls for bacteria. The diameters of the inhibition zones were calculated in millimeters.

##### 2.4.4.3 Determination of Minimum Inhibitory Concentration (MIC)

The MICs were determined using the microdilution method in 96‐well microtiter plates, as outlined by Negreiros et al. (2016) with some modifications. The EOs were serially diluted to achieve concentrations ranging from 1.125 to 144 μg mL^−1^. Then, 50 μL of each EO was combined with 100 μL of suited broth growth medium in the wells of a 96‐well microplate, followed by the addition of 10 μL of a standardized suspension of the organism to each well. The MIC values for gentamicin, ampicillin, nystatin, and penicillin were determined in balance trials to control the delicacy of the microorganisms.

#### 2.4.5 Isolation of EOs

The EOs were extracted from the samples through hydro‐distillation using a Clevenger apparatus, a process that lasted approximately 3 h. Post‐distillation, the EOs were collected in dark glass flasks and stored at 4°C until further analysis. The yield of EO was calculated employing the formula: EO yield^(%)^ = (mass of dry matter_(g)_ mass of EO_(g)_) × 100.

##### 2.4.5.1 Analysis of EO Constituents

The EOs were analyzed using Gas Chromatography (GC) on an Agilent 7890B system, which was equipped with a Flame Ionization Detector (FID) and an HP‐5 fused silica capillary column (30 m × 0.25 mm i.d.; 0.25 μm film thickness; J & W Scientific, Folsom). The oven temperature was programmed to rise from 60°C to 240°C at a rate of 3°C per min. The analysis was conducted with a split injection (split ratio 25:1), with the FID set at 265°C and the injector at 250°C. A 1 μL sample of the EO, diluted with hexane (1:10), was injected into the GC inlet, maintaining a column flow rate of 1 mL per min. For Gas Chromatography–Mass Spectrometry (GC–MS) analysis, the same Agilent gas chromatograph was used, coupled with a mass spectrometer detector (Model 5977 MSD) and an HP‐5 MS fused silica capillary column. Helium was used as the carrier gas, with an ionization voltage of 70 eV and an ion source temperature of 300°C. The scan time was set to 1 s, covering a mass range of 40–400 amu.

##### 2.4.5.2 Component Identification

The components of EOs were identified by comparing their retention indices (RI) with those of n‐alkanes (C5–C22) as reported in the literature. Further confirmation was achieved by fitting the noted mass spectra with those in the W9N11.L mass library of the GC–MS method and with documented mass spectra (Adams 2017).

#### 2.4.6 Extraction of Terpenoid Compounds

To quantitatively analyze terpenoid compounds, 10 mL of a methanol‐dichloromethane solvent mixture was combined with 0.5 g of the weighed samples. The samples were then subjected to ultrasonic extraction for 1 h. The supernatant was collected and refined through a 0.45 μm filter. The filtered samples were then tested using liquid chromatography–tandem mass spectrometry (LC–MS/MS) under specified modifications.

#### 2.4.7 Borneol, Camphor, and 1,8‐Cineole Profile Determination Using LC–MS/MS

Chromatographic separation was conducted using an ODS Hypersil C18 analytical column (150 × 2 mm, 4 μm, 80 Å, Phenomenex) from Thermo Finnigan (Dreieich, Germany). Terpenoids were separated into a binary solvent system consisting of 0.1% formic acid in water (solvent X) and 0.1% methanol (solvent Y). The mobile phase was returned to its initial condition and held for 4 min for system recalibration before the next injection. The flow rate was maintained at 0.2 mL min^−1^, with an injection volume of 10 μL and a column temperature of 30°C. The ion source was managed in positive electrospray ionization (ESI+) mode, with a spray needle voltage of 3.5 kV. The sheath and auxiliary gas flows were set at 5 and 35 arbitrary units, respectively. Source collision‐induced dissociation (CID) was set to 11 V, using argon as the collision gas at a pressure of 1.0 arbitrary units. During method development, borneol, camphor, and 1,8‐cineole were studied in full scan product mode, yielding m/z = 153 and 135 as the most prominent ions. Optimal areas were achieved with collision energies of 10 and 11 V, respectively. The tagged compounds (m/z = 174) produced corresponding ions at m/z = 156 and 138, using the same collision energy. For MS/MS analysis of borneol, camphor, and 1,8‐cineole, the mass transitions (m/z precursor ion/m/z product ion) were 171/135 and 171/153 for the unlabeled compounds, and 174/138 and 174/156 for the labeled compounds. The voltages applied to the precursor ions to generate the product ions m/z 135 or 138 and m/z 153 or 156 were 10 and 11 V, respectively. The peak width was set to 0.7 full width at half‐maximum, with a scan time of 0.2 s per transition and a scan width of ±0.7 amu. Reference standards of borneol, camphor, and 1,8‐cineole were obtained from Sigma‐Aldrich Co (St. Louis, MO, USA). These standards were reduced to create calibration standards with concentrations ranging from 1 to 200 μg mL^−1^. External calibration parameters and LC–MS/MS conditions, involving the calibration curve and correlation coefficient (*R* ^2^), were established.

#### 2.4.8 Statistical Analysis

The Levene test was used to assess the equality of variances among all values. Subsequently, One‐Way Analysis of Variance (ANOVA) was performed using SAS‐JMP software (version 10). Post hoc comparisons of means were carried out with the LSD test at a 95% confidence level, and the findings are presented as mean values with standard deviations. To explore the relationships between various parameters, principal component analysis was utilized. Pearson's correlation coefficients between the compounds and antioxidant capabilities were calculated using version 26 SPSS (Chicago, USA) software.

## 3 Results and Discussion

### 3.1 Essential Oil (EO) Yield (% w/w) and EO Composition

The data on EO yield and components from *A. gypsicola* treated with SA and CTS are detailed in Table 2. Both treatment methods resulted in an increase in EO yield compared to the control. The highest values were obtained from the 2 mM SA and 4 g L^−1^ CTS treatments, with increases of 40% and 34.3%, respectively. While EO yield continued to increase with 8 mM SA and 8 g L^−1^ CTS applications compared to the control, it was found that the yield increase trend ended. These fluctuations in yield highlight the impact of SA and CTS doses on the essential oil production of yarrow. In previous studies, the oil yield of *A. gypsicola* from Turkey was reported to be 0.65% and 1.20% (Açıkgöz 2019). Nevertheless, this yield showed significant variability, ranging from 0.60% to 1.20% based on the ontogenetically, morphogenetically, and diurnally (Açıkgöz 2020). Additionally, Other studies on *Achillea* species have reported yields ranging from 0.05% to 2.70% (Mohammadhosseini et al. 2017; Amssayef et al. 2024; Vojoudi et al. 2024). Although there are many studies that SA and CTS applications increase the yield of essential oil (El‐Ziat et al. 2024; Medeiros et al. 2024), some researchers have suggested that these applications do not affect the yield of essential oil (Pirbalouti et al. 2019). In this study, important enhancements in EO yield were detected in both applications. The EO compositions of *A. gypsicola* are given in Table 2. According to the analysis of variance, there was a significant change in the chemical compositions of EOs from SA and CTS treatments. Twenty‐nine compounds were identified by the analysis of EOs compositions. The proportions of these compounds varied between 96.9% and 98.63% in the oils obtained from this study. Borneol, camphor, 1,8‐cineole, alcohol, borneol, terpinen‐4‐ol, lavandulol, and cis‐4‐thujanol were the main components, but the percentages of these compounds in the EOs varied depending on the SA and CTS treatments. As a result of SA and CTS treatments, the 1,8‐cineole ratio varied between 14.52% and 40.53%, while the highest ratio was obtained from CTS 4 g L^−1^ treatment. In SA 0.5 mM treatment, the 1,8‐cineole ratio decreased compared to the control. In general, an increase of 87.5% in 1,8‐cineole was obtained in the CTS 4 g L^−1^ treatment and 26.9% in the SA 8 mM treatment. The camphor compound responded positively to all treatments and increased in rate. The prominent treatments here were 0.5‐ and 2‐mM doses of SA, which provided an increase of 1.24 and 0.78‐fold, respectively. Borneol did not respond positively to any of the treatments and decreased significantly compared to the control group. Similarly, compounds such as lavandulol, cis‐4‐thujanol, and terpinen‐4‐ol also decreased in percentage with the treatments. Previous studies have reported that *A. gypsicola* EOs mostly contain cis‐4‐thujanol, 1,8‐cineole, terpinen‐4‐ol, camphor, and borneol. Also, in these studies, camphor (44%), cis‐4‐thujanol (5%), mentacamphor (20%), menthone (23%) and verbenol <trans‐>(6%) were synthesized in high amounts during the before flowering period; menthol—18.38%, 1,8‐cineole—49%, and terpinene—4% were synthesized in high amounts during the full flowering period; borneol (23%), terpinen <γ‐>(11%) and verbenone (6%) were synthesized in high amounts during the post‐flowering period. However, Studies have shown that the concentrations of compounds such as camphor, cineole, borneol, menthone, menthol, and mentacamphor are different on the harvest times. For example, while the accumulation of 1,8‐cineole was the highest in the pre‐flowering period (27%), camphor accumulation was the highest in the post‐flowering period (Açıkgöz 2019, 2020). Then again, borneol accumulation was the maximum in the full flowering period. Studies confirm that these proportional changes in the essential oils of medicinal and aromatic plants are affected by many factors such as harvest times and phenological stages (Açıkgöz 2020) genetics (Sharma et al. 2019), environmental conditions (Pant et al. 2021), and pre‐ and after harvest processes (Ay et al. 2023a, 2023b). Considering these factors, it may be possible to increase the plant quality by increasing the volatile or non‐volatile bioactive compounds of plants. Previous studies have reported high abundances of certain compounds in species belonging to the genus *Achillea*. For instance, 1,8‐cineole (45.2%), ascaridole (43.22%), and iso‐ascaridole (37.87%) were identified as main compounds in *Achillea biebersteinii* (Mirahmadi and Norouzi 2017). In *A. kellalensis* and *A. wilhelmsii*, lavandulyl acetate (26.19%) and chamazulene (52.60%) were the predominant compounds, respectively (Ghasemi Pirbalouti 2017). *A. wilhelmsii* also contained significant amounts of camphor, 1,8‐cineole, anethole, and α‐pinene. For *A. vermicularis* , the main compounds were 1,8‐cineole, camphor, levo‐carvone, and δ‐terpinene, while *A. tenuifolia* was rich in β‐cubebene, elixene, β‐sesquiphellandrene, 1,8‐cineole, camphor, and δ‐terpinene (Farajpour et al. 2024). In *A. fragrantissima*, artemisia ketone (22.11%) and β‐thujone (31.86%) were the main components (Alsohaili 2018). *A. wilhelmsii* was also found to contain neoiso‐dihydrocarveol acetate (25.2%), trans‐piperitol (11.7%), trans‐carveol (27.5%), chrysanthenone (38.8%), filifolone (19.7%), α‐pinene (11.8%), and (E)‐nerolidol (10.8%), (E)‐caryophyllene (11.2%) (Saeidi et al. 2018). Studies on *A. gypsicola* are limited, and most have been conducted on wild samples. This study is the first to investigate cultivated samples, identifying camphor (40.17%–43.53%), 1,8‐cineole (22.01%–49.36%), and borneol (9.50%–22.62%) as the main compounds (Açıkgöz 2019, 2020).

**TABLE 2:** Composition of essential oils (%) of whole plant depending on the full flowering stage in *A. gypsicola* (mean of 2 years).

No	Compounds	RI	Treatments							
			Control (water + ethanol)	SA 0.5 mM	SA 2 mM	SA 8 mM	Control (water + ethanol)	CTS 2 g L^−1^	CTS 4 g L^−1^	CTS 8 g L^−1^
	Essential oil yield (%)		0.70^d^ ± 0.08	0.85^c^ ± 0.10	0.98^a^ ± 0.09	0.90^b^ ± 0.02	0.70^d^ ± 0.05	0.78^c^ ± 0.05	0.94^a^ ± 0.10	0.88^b^ ± 0.05
1	Pentanal	706	0.04 ± 0.01	0.05 ± 0.00	0.04 ± 0.01	0.05 ± 0.02	0.04 ± 0.00	0.03 ± 0.00	0.05 ± 0.00	0.05 ± 0.00
2	Menthene < 1‐ρ‐>	1026	0.60 ± 0.05	0.67 ± 0.08	0.58 ± 0.08	0.59 ± 0.05	0.60 ± 0.05	0.31 ± 0.07	0.32 ± 0.08	0.39 ± 0.05
3	**1,8‐Cineole**	**1031**	**21.60 ± 0.32** ^**b**^	**14.52 ± 0.57** ^**c**^	**21.85 ± 0.09** ^**b**^	**27.36 ± 0.09** ^**a**^	**21.60 ± 0.32** ^**b**^	**35.55 ± 1.18** ^**b**^	**40.53 ± 0.80** ^**a**^	**29.54 ± 0.16** ^**c**^
4	α‐Tolualdehyde	1042	0.11 ± 0.03	0.15 ± 0.01	0.16 ± 0.02	0.13 ± 0.00	0.11 ± 0.01	0.22 ± 0.02	0.19 ± 0.02	0.24 ± 0.00
5	2‐Formylphenol	1044	0.14 ± 0.00	0.17 ± 0.01	0.13 ± 0.02	0.14 ± 0.03	0.14 ± 0.03	0.30 ± 0.02	0.33 ± 0.00	0.32 ± 0.00
6	Terpinene <γ‐>	1059	2.17 ± 0.12	1.25 ± 0.10	1.28 ± 0.20	1.27 ± 0.17	2.17 ± 0.09	1.30 ± 0.12	1.25 ± 0.10	1.26 ± 0.18
7	**Cis‐4‐thujanol**	**1098**	**4.77 ± 0.28** ^**a**^	**3.01 ± 0.30** ^**c**^	**2.65 ± 0.25** ^**e**^	**3.33 ± 0.42** ^**b**^	**4.77 ± 0.40** ^**a**^	**2.86 ± 0.30** ^**d**^	**2.90 ± 0.20** ^**d**^	**2.82 ± 0.38** ^**d**^
8	Menth‐2‐en‐1‐ol <trans‐ρ‐>	1140	0.30 ± 0.09	0.22 ± 0.03	0.28 ± 0.04	0.27 ± 0.01	0.30 ± 0.01	0.36 ± 0.00	0.28 ± 0.02	0.22 ± 0.00
9	Verbenol <trans‐>	1144	0.38 ± 0.01	0.25 ± 0.00	0.24 ± 0.00	0.28 ± 0.00	0.38 ± 0.01	0.30 ± 0.00	0.25 ± 0.02	0.18 ± 0.00
10	**Camphor**	**1146**	**22.02 ± 1.10** ^**d**^	**49.28 ± 1.64** ^**a**^	**39.20 ± 0.75** ^**b**^	**32.10 ± 0.22** ^**c**^	**22.02 ± 0.65** ^**c**^	**23.92 ± 0.38** ^**b**^	**24.02 ± 0.90** ^**b**^	**32.00 ± 1.10** ^**a**^
11	Menthone	1152	0.25 ± 0.02	0.29 ± 0.05	0.42 ± 0.07	0.33 ± 0.05	0.25 ± 0.04	0.23 ± 0.01	0.24 ± 0.09	0.27 ± 0.01
12	Pinocarvone	1164	0.13 ± 0.02	0.09 ± 0.01	0.10 ± 0.04	0.10 ± 0.07	0.13 ± 0.03	0.11 ± 0.01	0.19 ± 0.07	0.25 ± 0.01
13	**Borneol**	**1169**	**23.22 ± 0.60** ^**a**^	**15.74 ± 0.70** ^**c**^	**16.80 ± 0.46** ^**b**^	**16.56 ± 0.46** ^**b**^	**23.22 ± 0.60** ^**a**^	**15.98 ± 0.74** ^**c**^	**16.92 ± 0.18** ^**b**^	**19.85 ± 0.20** ^**a**^
14	**Lavandulol**	**1169**	**4.52 ± 0.12** ^**a**^	**3.85 ± 0.52** ^**b**^	**3.68 ± 0.24** ^**c**^	**3.70 ± 0.11** ^**c**^	**4.52 ± 0.12** ^**a**^	**2.86 ± 0.10** ^**d**^	**2.99 ± 0.30** ^**d**^	**2.88 ± 0.08** ^**d**^
15	Menthacamphor	1171	2.17 ± 0.23	1.12 ± 0.17	1.13 ± 0.00	2.09 ± 0.22	2.17 ± 0.00	2.15 ± 0.00	1.16 ± 0.00	1.17 ± 0.20
16	Menthol	1171	2.69 ± 0.33	1.72 ± 0.27	1.67 ± 0.03	1.70 ± 0.27	2.69 ± 0.03	0.78 ± 0.03	0.80 ± 0.03	0.77 ± 0.03
17	**Terpinen‐4‐ol**	**1177**	**4.44 ± 0.30** ^**a**^	**0.68 ± 0.10**	**1.00 ± 0.50** ^**e**^	**1.00 ± 0.10** ^**e**^	**4.44 ± 0.30** ^**a**^	**3.02 ± 0.32** ^**b**^	**1.42 ± 0.24** ^**d**^	**1.65 ± 0.30** ^**c**^
18	Terpineol <α‐>	1188	0.18 ± 0.01	0.28 ± 0.08	0.27 ± 0.01	0.27 ± 0.09	0.18 ± 0.01	0.35 ± 0.00	0.25 ± 0.01	0.44 ± 0.09
19	Verbenone	1205	1.57 ± 0.12	0.83 ± 0.09	0.72 ± 0.02	0.70 ± 0.12	1.57 ± 0.05	0.78 ± 0.02	0.96 ± 0.02	1.05 ± 0.02
20	Pulegone	1237	0.70 ± 0.10	1.03 ± 0.01	1.08 ± 0.02	0.67 ± 0.10	0.70 ± 0.11	1.73 ± 0.02	1.10 ± 0.02	0.95 ± 0.05
21	Carvotanacetone	1247	0.25 ± 0.01	0.21 ± 0.00	0.19 ± 0.02	0.22 ± 0.04	0.25 ± 0.03	0.18 ± 0.03	0.27 ± 0.02	0.25 ± 0.05
22	Thymoquinone	1252	0.67 ± 0.00	0.30 ± 0.00	0.32 ± 0.00	0.32 ± 0.02	0.67 ± 0.00	0.30 ± 0.07	0.33 ± 0.00	0.30 ± 0.00
23	Piperitone	1252	0.14 ± 0.00	0.12 ± 0.01	0.12 ± 0.01	0.14 ± 0.01	0.14 ± 0.00	0.09 ± 0.00	0.10 ± 0.01	0.14 ± 0.02
24	Isobornyl acetate	1285	0.11 ± 0.00	0.08 ± 0.00	0.07 ± 0.01	0.10 ± 0.02	0.11 ± 0.00	0.12 ± 0.00	0.12 ± 0.01	0.12 ± 0.03
25	Eugenol	1359	1.19 ± 0.01	1.31 ± 0.25	1.40 ± 0.01	1.75 ± 0.21	1.19 ± 0.14	0.42 ± 0.00	0.26 ± 0.01	0.43 ± 0.01
26	Caryophyllene <(E)‐>	1419	0.15 ± 0.01	0.10 ± 0.01	0.12 ± 0.01	0.13 ± 0.03	0.15 ± 0.01	0.09 ± 0.02	0.10 ± 0.01	0.11 ± 0.02
27	Germacrene D	1481	2.85 ± 0.01	1.08 ± 0.03	0.98 ± 0.01	2.05 ± 0.15	2.85 ± 0.33	1.88 ± 0.02	0.15 ± 0.01	0.28 ± 0.01
28	Selinene <β‐>	1490	0.17 ± 0.01	0.15 ± 0.05	0.14 ± 0.01	0.17 ± 0.05	0.17 ± 0.03	0.14 ± 0.03	0.12 ± 0.00	0.12 ± 0.01
	Eudesmol <β‐>	1650	0.25 ± 0.01	0.33 ± 0.07	0.32 ± 0.01	0.30 ± 0.01	0.25 ± 0.03	0.51 ± 0.03	0.62 ± 0.08	0.58 ± 0.01
	All identified components		97.78	98.9	96.9	97.82	97.78	97.17	98.2	98.63

*Note:* Bold value indicates statistically significant difference.

^a^ SA, Salicylic acid.

^b^ CTS, Chitosan.

^c^ Retention indices relative to n‐alkans (C5‐C22) on HP 5MS column.

^d^ The result are expressed as means ± SD (*n* = 3).

^e^ Means with similar letter in % 5 level of LSD test are not significant.

Research has shown that stimulation with SA and CTS enhances the composition and production of EOs in medicinal and aromatic plants (Silva‐Santos et al. 2023). SA initiates the synthesis of SMs such as essential oils, proteins, and phenolic compounds, with responses varying based on the plant species, dose, and environmental conditions (Miladinova‐Georgieva et al. 2022; Ay et al. 2023b; Jeyasri et al. 2023; Kırgeç et al. 2023). Chitosan, a biotic elicitor, has been shown to alter the composition of EOs in *Lippia alba* and promote the synthesis of specific compounds (de Souza Silva et al. 2022). Elicitors, both biotic and abiotic, trigger physiological and morphological responses in plants. For example, chitosan application significantly increased vestitol content in the leaves of *Lotus japonicus* (Trush et al. 2023). The synergistic effect of SA and chitosan on SM production has been observed, with greater increases in stevioside and rebaudioside A levels in *Stevia rebaudiana* compared to control (Nawaz et al. 2023).

Plants have receptors that recognize external signals and activate defense systems to synthesize bioactive compounds through secondary metabolism. However, MAP species often do not accumulate sufficient amounts of bioactive molecules, necessitating stimulation to enhance secondary metabolism and increase SM content. Studies have shown that chitosan increases the proportions of menthol and menthone (Ahmad et al. 2019), as well as citronellal and geranial compounds in EOs (Ahmed et al. 2020). SA application has been found to increase the concentrations of main monoterpenes in peppermint EO (Cappellari et al. 2019). Chitosan, a natural biopolymer, stimulates the biosynthesis of SA and methyl jasmonate, enhancing plant physiological responses through stress transduction signaling pathways (Açıkgöz 2019, 2020). The foliar practice of SA importantly enhanced the content of oxygenated sesquiterpenes and sesquiterpene hydrocarbons, although it did not affect the proportions of some compounds in the EO (Medeiros et al. 2024).

### 3.2 AntiMicrobial Screening

The antimicrobial activity of *A. gypsicola* EOs was evaluated, including three Gram‐negative bacteria, four Gram‐positive bacteria, one yeast, and two fungi (Table 3). Significant inhibitory effects were observed in *E. coli* (21.2 mm), *P. aeruginosa* (23.4 mm), and *S. aureus* (28.3 mm) with the CTS 8 g L^−1^ treatment. The highest antimicrobial activity was recorded in *B. subtilis* (28.8 mm) with CTS 8 g L^−1^, followed by CTS 4 g L^−1^ (28.1 mm) and CTS 2 g L^−1^ (25.8 mm). For *S. faecalis* , *L. monocytogenes* , and *S. cerevisiae* , the CTS 8 g L^−1^ treatment yielded the highest activity (24.6, 29.2, and 19.5 mm, respectively), with CTS 4 g L^−1^ also showing significant results (21.8, 26.8, and 18.1 mm, respectively). The highest activity against *C. albicans* and *A. niger* was observed with SA 2 mM (19.8 and 19.2 mm, respectively). These outcomes were notably higher compared to water + ethanol. The CTS 8 g L^−1^ treatment demonstrated a larger inhibition zone (21.2 mm) against *E. coli* compared to ampicillin. Similarly, CTS 8 g L^−1^ treatments showed superior results against *P. aeruginosa* and *S. aureus* (23.4 and 28.3 mm, respectively) compared to gentamicin (23 and 28 mm, respectively). The minimum inhibitory concentrations (MIC) of *A. gypsicola* EOs ranged from 4.5 to 72 μg mL^−1^ for Gram‐positive bacteria, 4.5–144 μg mL^−1^ for Gram‐negative bacteria, and 18–144 μg mL^−1^ for fungi. The most effective MIC values were observed in CTS 2 and 4 g L^−1^ treatments for *P. vulgaris* (4.5 μg mL^−1^), *S. aureus* (4.5 μg mL^−1^), and *B. subtilis* (4.5 μg mL^−1^). Other treatments did not perform as well compared to the positive control. Numerous studies have highlighted the antimicrobial properties of *Achillea* species.

**TABLE 3:** Inhibition zone (IZ) and minimum inhibitory concentration (MIC) of essential oils belonging to whole plant depending on the full flowering stage in *A. gypsicola* (mean of 2 years).

Treatments		Bacterial, yeast and fungal strain									
		Gram‐negative bacteria			Gram‐positive bacteria				Yeast strain	Fungal strain	
		*Escherichia coli*	*Proteus vulgaris*	*Pseudomonas aeruginosa*	*Staphylococcus aureus*	*Bacillus subtilis*	*Streptococcus faecalis*	*Listeria monocytogenes*	*Saccharomyces cerevisiae*	*Candida albicans*	*Aspergillus niger*
Control (water + ethanol)	IZ (mm)	15.5 ± 0.12	16.5 ± 0.20	16.1 ± 0.50	17.5 ± 0.14	18.4 ± 0.70	13.2 ± 0.35	14.3 ± 0.07	9.1 ± 0.12	10.8 ± 0.15	11.2 ± 0.10
	MIC (μg mL^−1^)	144 ± 0.15	36 ± 0.20	36 ± 0.40	18 ± 0.68	9 ± 0.05	36 ± 0.16	36 ± 0.10	144 ± 0.00	72 ± 0.70	72 ± 0.36
SA 0.5 mM	IZ (mm)	14.4 ± 0.12	17.2 ± 0.10	18.4 ± 0.00	16.5 ± 0.32	20.4 ± 0.20	12.8 ± 0.40	16.2 ± 0.10	12.6 ± 0.30	13.8 ± 0.80	17.5 ± 0.32
	MIC (μg mL^−1^)	72 ± 0.20	36 ± 0.12	36 ± 0.34	9 ± 0.05	9 ± 0.05	18 ± 0.20	36 ± 0.50	72 ± 0.40	72 ± 0.11	36 ± 0.15
SA 2 mM	IZ (mm)	15.3 ± 0.17	20.4 ± 0.10	17.2 ± 0.10	18.6 ± 0.20	21.5 ± 0.15	17.9 ± 0.93	14.1 ± 0.42	14.4 ± 0.17	19.8 ± 0.13	19.2 ± 0.04
	MIC (μg mL^−1^)	36 ± 0.10	36 ± 0.10	18 ± 0.12	18 ± 0.00	9 ± 0.00	18 ± 0.00	36 ± 0.12	72 ± 0.65	72 ± 0.65	72 ± 0.30
SA 8 mM	IZ (mm)	17.2 ± 0.51	20.5 ± 0.30	16.8 ± 0.00	16.2 ± 0.12	20.6 ± 0.11	18.9 ± 0.13	16.4 ± 0.40	12.5 ± 0.20	13.5 ± 0.10	18.6 ± 0.20
	MIC (μg mL^−1^)	18 ± 0.02	18 ± 0.05	36 ± 0.18	18 ± 0.10	2.25 ± 0.12	36 ± 0.80	144 ± 0.15	18 ± 0.12	36 ± 0.10	36 ± 0.36
CTS 2 g L^−1^	IZ (mm)	17.2 ± 0.30	22.6 ± 0.10	18.5 ± 0.20	22.3 ± 0.11	25.8 ± 0.60	20.5 ± 0.01	24.1 ± 0.30	16.8 ± 0.35	13.3 ± 0.44	13.6 ± 0.20
	MIC (μg mL^−1^)	72 ± 0.05	4.5 ± 0.09	18 ± 0.00	9 ± 0.20	4.5 ± 0.00	9 ± 0.18	144 ± 0.30	36 ± 0.00	36 ± 0.15	36 ± 0.07
CTS 4 g L^−1^	IZ (mm)	16.3 ± 0.25	23.8 ± 0.30	21.1 ± 0.18	26.6 ± 0.15	28.1 ± 0.50	21.8 ± 0.08	26.8 ± 0.10	18.1 ± 0.20	12.8 ± 0.42	14.3 ± 0.10
	MIC (μg mL^−1^)	18 ± 0.56	4.5 ± 0.05	9 ± 0.13	4.5 ± 0.00	4.5 ± 0.10	9 ± 0.30	36 ± 0.52	18 ± 0.10	72 ± 0.90	72 ± 0.20
CTS 8 g L^−1^	IZ (mm)	21.2 ± 0.09	16 ± 0.10	23.4 ± 0.24	28.3 ± 0.25	28.8 ± 0.15	24.6 ± 0.18	29.2 ± 0.30	19.5 ± 0.00	12.5 ± 0.20	10.8 ± 0.30
	MIC (μg mL^−1^)	18 ± 0.10	36 ± 0.25	9 ± 0.09	18 ± 0.05	18 ± 0.22	72 ± 0.00	72 ± 0.36	72 ± 0.18	36 ± 0.20	18 ± 0.10
Ampicillin^a^	IZ (mm)	18 ± 0.10	38 ± 0.10	nt	nt	30 ± 0.15	13 ± 0.15	nt	nt	nt	nt
	MIC (μg mL^−1^)	4 ± 0.10	8 ± 0.15	nt	nt	0.5 ± 0.01	2 ± 0.30	nt	nt	nt	nt
Gentamicin^a^	IZ (mm)	nt	nt	23 ± 0.05	28 ± 0.06	44 ± 0.15	nt	13 ± 0.30	nt	nt	nt
	MIC (μg mL^−1^)	nt	nt	8 ± 0.45	8 ± 0.10	4 ± 0.18	nt	1 ± 0.05	nt	nt	nt
Nystatin^b^	IZ (mm)	nt	nt	nt	nt	nt	nt	nt	25 ± 0.50	42 ± 0.54	40 ± 0.30
	MIC (μg mL^−1^)	nt	nt	nt	nt	nt	nt	nt	1 ± 0.10	2 ± 0.54	8 ± 0.07
Penicillin^a^	IZ (mm)	nt	nt	nt	27 ± 0.80	nt	nt	nt	nt	nt	nt
	MIC (μg mL^−1^)	nt	nt	nt	0.5 ± 0.03	nt	nt	nt	nt	nt	nt

*Note:* nt, not tested.

^a^ Diameter of zone of inhibition (mm) including disk diameter of 6 mm.

^b^ Tested at 10 μg disc^−1^.

The common point of these studies is not cultural practices, but the antimicrobial activity tests of raw materials obtained from nature, either extracts or essential oils. We know from the studies that cultural practices on plants improve and develop the quality of plant extracts or EOs (Ay et al. 2023a, 2023b; Kırgeç et al. 2023). Therefore, expanding and investigating these practices may be an important step against microorganisms that develop themselves day by day. The focus of this study is exactly this and to improve the content of SMs that constitute an important defense against microorganisms with SA and CTS treatments. It is the rapid and short‐term release of reactive oxygen species (ROS), which is one of the early defense reactions against micro or macro‐organisms, and these include a number of defense reactions. In numerous studies conducted so far, researchers have indicated that SA and chitosan elicitors support the accumulation of SMs synthesized in plants (Cui et al. 2024; Novikova et al. 2024). Moreover, it is known that chitosan can inhibit bacterial (Cheng et al. 2022) growth by chelating metal ions, and that salicylaldehyde amino acid Schiff bases also possess chelating sites (Odularu 2022). On the contrary, it has been confirmed in many studies that these compounds contribute to the development of antimicrobial potential by increasing ROS activity (Açıkgöz et al. 2024). Lal et al. (2016) reported that Schiff‐based chitosan exhibited inhibitory effects against certain fungal species, including *A. niger* , as well as gram‐positive bacteria such as *B. subtilis* and *S. aureus* . In another study, Piegat et al. (2020) demonstrated that N‐O acylated chitosan derivatives possessed antibacterial activity against *E. coli* and *S. aureus* using microdilution, disk diffusion, and agar immersion methods. Similarly, in our findings, CTS treatments were prominently featured. Attia et al. (2018) highlighted that salicylic acid treatments are effective against *S. aureus* , *P. aeruginosa* , *E. coli* , *K. pneumoniae* , and *Candida* species, while Song et al. (2022) confirmed the antibacterial efficacy of salicylic acid applications. The varying antimicrobial properties of plant extracts or essential oils against microorganisms can be attributed to the differing levels at which these compounds are combined. This variability can lead to compounds exhibiting stronger or weaker antimicrobial effects, influenced by their antagonistic or synergistic interactions (Açıkgöz 2020). It should also be noted that these compounds vary based on vegetative stages (Ay et al. 2023a; Kırgeç et al. 2023). Therefore, it is crucial to enrich these compounds, which differ according to many variables, with natural and endogenous stimulants to combat increasingly resistant microorganisms without excessively polluting the soil.

### 3.3 Total Flavonoid and Phenolic Contents

The total flavonoid (TF) and phenolic (TP) contents gained from samples treated with SA and CTS at the beginning of the flowering period are presented in Table 4. The total phenolic content (TPC) values exhibited a significant enhancement referred to the control group, hanging on the compound and dose used. The TPC ranged from 330.4 to 468.1 mg GAE g^−1^ DW, with the highest value observed in the SA 2 mM treatment (468.1 mg GAE g^−1^ DW), compared to the control (330.4 mg GAE g^−1^ DW). Specifically, TPC increased by 45.4% and 30.2% in the SA 2 mM and SA 8 mM treatments, respectively, compared to the control. Although significant increases in TPC were also observed in the CTS treatments, the SA treatments were more effective. Conversely, the CTS treatments resulted in higher TFC values, with significant increases compared to the control group. The TFC ranged from 98.2 to 175.5 mg QE g^−1^ DW, with the highest values recorded in the CTS 8 g L^−1^ and 4 g L^−1^ treatments (175.5 and 159.3 mg QE g^−1^ DW, respectively). The TFC increased by 78.7% with the CTS 8 g L^−1^ treatment compared to the control. While SA treatments also significantly increased TFC values compared to the control, they were slightly less effective than the CTS treatments. Generally, the TPC and TFC of *Achillea* species vary depending on the variety/species, plant parts, developmental stages, and cultural practices (Binobead and Aziz 2024; Bouteche et al. 2024; Kbaydet et al. 2024; Raudone et al. 2024; Siles‐Sánchez et al. 2024). The highest increase in TPC was observed with the 2 mM SA dose. Therefore, to enhance these properties in *A. gypsicola*, a 2 mM SA application and an 8 g L^−1^ CTS treatment are recommended. SA can be moved from the treatment site to other plant tissues, fulfilling its functions in externally used SA applications (Schweiger et al. 2014; Mozafari et al. 2018). SA, organic procured cinnamic acid, acts as an intercede in the shikimic acid pathway and acts as a role in the synthesis of phenolics (Hayat et al. 2007). Phenols are combined via the shikimic acid metabolic pathway. The enhancing effect of SA on phenolic compounds in *A. gypsicola* may be attributed to this typical. SA is an organic compound that functions as a plant growth regulator (Kaya et al. 2023).

**TABLE 4:** Effects of salicylic acid (SA) and chitosan (CTS) applied to *A. gypsicola* as a foliar spray at different doses at the full flowering stage on total phenolic (mg GAE g^−1^ DW), flavonoids (mg QE g^−1^ DW), antioxidant activity (% inhibition), borneol, camphor, and 1,8‐cineole (μg mL^−1^) (mean of 2 years).

	Treatments	Total phenolic content (mg GAE g^−1^ DW)	Total flavonoid content (mg QE g^−1^ DW)	DPPH radical scavenging assay (%)*	Ferrous ions chelating assay (%)*
	Control (water + ethanol)	330.4 ± 1.8^f^	98.20 ± 0.8^g^	65.9 ± 0.9^f^	57.1 ± 2.2^f^
	SA 0.5 mM	388.5 ± 2.3^c^	112.0 ± 0.3^f^	69.4 ± 1.2^d^	66.9 ± 1.4^d^
	SA 2 mM	468.1 ± 1.5^a^	110.5 ± 0.8^e^	70.1 ± 0.7^d^	67.2 ± 2.5^d^
	SA 8 mM	430.1 ± 1.4^b^	126.5 ± 0.8^d^	67.7 ± 0.8^e^	64.9 ± 1.6^e^
	CTS 2 g L^−1^	390.5 ± 3.7^c^	137.5 ± 0.4^c^	76.6 ± 0.3^c^	70.5 ± 1.0^c^
	CTS 4 g L^−1^	357.2 ± 2.6^e^	159.3 ± 0.9^b^	89.5 ± 1.0^b^	79.5 ± 1.9^b^
	CTS 8 g L^−1^	376.5 ± 3.7^d^	175.5 ± 0.5^a^	93.8 ± 0.6^a^	85.7 ± 3.2^a^
	EDTA	—	—	nt	97.6 ± 1.3
Positive control	BHT	—	—	83.3 ± 0.8	96.3 ± 1.2
	Trolox	—	—	85.6 ± 0.9	97.8 ± 0.6
	Treatments	Borneol (μg mL^−1^)	Camphor (μg mL^−1^)	1,8‐Cineole (μg mL^−1^)	
	Control (water + ethanol)	37.20 ± 1.10^e^	63.40 ± 0.8^f^	78.10 ± 1.1^f^	
	SA 0.5 mM	49.13 ± 0.32^c^	148.30 ± 0.3^d^	85.70 ± 1.8^e^	
	SA 2 mM	60.19 ± 0.65^a^	186.52 ± 0.8^b^	114.5 ± 1.4^d^	
	SA 8 mM	38.10 ± 1.40^e^	114.28 ± 1.4^g^	128.3 ± 1.4^b^	
	CTS 2 g L^−1^	43.35 ± 0.34^d^	137.34 ± 0.4^e^	122.3 ± 0.8^c^	
	CTS 4 g L^−1^	55.80 ± 0.21^b^	165.10 ± 0.9^c^	175.8 ± 2.9^a^	
	CTS 8 g L^−1^	48.60 ± 0.07^c^	198.60 ± 0.5^a^	128.7 ± 1.7^b^	

^a^ *At 200 μg mL^−1^ concentration.

^b^ DPPH, 2,2‐diphenyl‐1‐picrylhydrazyl.

^c^ nt, not tested.

^d^ SA, Salicylic acid.

^e^ CTS, Chitosan.

^f^ The results are expressed as means ± SD (*n* = 3).

^g^ Means with similar letter in % 5 level of LSD test are not significant.

### 3.4 Antioxidant Activity

Examining the antioxidant capacity of *A. gypsicola* plants used with SA and CTS is crucial for assessing the therapeutic efficacy of these samples. The antioxidant capacity was evaluated using DPPH and Ferrous ion chelation tests, with results expressed in Table 4. The study found a strong correlation between the results of the two antioxidant capacity assays. Treatments with CTS (8 g L^−1^, 93.8%) significantly increased antioxidant activity compared to the control group (65.9%) (Table 4). Additionally, all treatments exhibited statistically significant radical scavenging activity. The findings indicated that SA and CTS treatments enhanced radical scavenging capacity. Furthermore, these treatments demonstrated higher antioxidant capacity than positive controls like Trolox and BHT. Samples treated with CTS (8 g L^−1^) showed the highest iron ion chelation power (85.7%), followed by CTS (4 g L^−1^) with 79.5% chelation ability. Overall, the chelation ability ranked as Trolox > EDTA > BHT > CTS (8 g L^−1^) (Table 4). The relationship between SA and CTS treatments and SMs, as well as their effects on antioxidant activity, has been highlighted in numerous previous studies (Ahmed et al. 2024; Angouti et al. 2024). Stasińska‐Jakubas et al. (2024) reported that SA applications increased ROS activity, thereby enhancing antioxidant capacity in their study on *Hypericum perforatum* . Similarly, Rithichai et al. (2024) found that a 1 mM SA treatment resulted in the highest antioxidant capacity in *Ocimum sanctum* , while 2‐ and 2.5‐mM SA treatments led to a decrease in antioxidant capacity. This study demonstrates that SA and CTS stimulants can enhance antioxidant capacity. Additionally, several studies have emphasized that the use of SA and CTS treatments, either individually or in combination, supports the accumulation of SMs that are effective in improving antioxidant capacity (Das et al. 2024; Tabassum et al. 2024; Wang et al. 2024; Yin et al. 2024). The results of this study are consistent with current literature. The findings from this study highlight the significant effects of SA and CTS treatments on *A. gypsicola* and suggest that these stimulants can potentially increase antioxidant activity.

### 3.5 Terpenoid Profile and Determinations of Correlations Between the Obtained Results Using Pearson's Correlation and Principal Component Analysis (PCA)

The quantification of borneol, camphor, and 1,8‐cineole was conducted using LC–MS/MS analysis, and the results were stated as μg mL^−1^. The effects of SA and CTS applications used on *A. gypsicola* for the collection of terpenoids are exhibited in Table 4. Hence, the highest contents of borneol, camphor, and 1,8‐cineole were obtained from SA 2 mM, CTS 8 g L^−1^, and CTS 8 g L^−1^ treatments, with an increase in 0.62‐fold (from 37.20 to 60.19 μg mL^−1^), 2.13‐fold (from 63.4 to 198.6 μg/mL), and 1.25‐fold (from 78.1 to 175.8 μg/mL), respectively (Table 4). The level of borneol increased in SA 0.5‐ and 2‐mM treatments compared to the control group, and this increase stopped with SA 8 mM treatment and was in the same statistical group as the control group. According to these results, the 8 mM treatment can be seen as the limit value. Contrary to this situation, no limit dose was reached in camphor and 1,8‐cineole, and the increase in the amount of both compounds continued at different doses. While CTS was the prominent treatment in the amount of camphor and 1,8‐cineole among all treatments, SA treatment was the dominant treatment in the borneol compound. In previous studies conducted on borneol, camphor, and 1,8‐cineole terpenoids, researchers generally focused on their percentage ratios in essential oils (El‐Esawi et al. 2017; Gorni et al. 2020). Es‐sbihi et al. (2020) stated in their study on *Salvia officinalis* that SA treatment increased the camphor and 1,8‐cineole ratio in the essential oil. In another study, Abbaszadeh et al. (2020) reported that SA treatment in *Rosmarinus officinalis* improved the proportions of borneol, camphor, and 1,8‐cineole in essential oils. Similarly, Khodadadi et al. (2022) reported in their study on *Salvia abrotanoides* and *Salvia yangii* that CTS treatment increased the proportions of these compounds in the essential oil under stress conditions. Therefore, there are very few studies investigating the changes in the amount of these internal terpenes due to SA and CTS treatments. In his study on cell suspension cultures in *A. gypsicola*, Açıkgöz (2017) reported that the highest camphor production from SA and CTS stimulants was in CTS‐treated samples. The factor loadings from the PCA are exhibited in Table 5. PCA_1_ described 40.20% data variation and modification of the samples by the contents of cis‐4‐thujanol, lavandulol, terpinen‐4‐ol essential oil (+), and essential oil yield (+). PCA_2_ explained 26.53% of the variability in the original responses based on 1,8‐cineole essential oil (+), 1,8‐cineole content (+), TPC (+), and TFC (+). PCA_3_ explained 15.43% of the variability in the original responses based on borneol essential oil (+), borneol content (+), DPPH radical scavenging (+), and Ferrous ions chelating assay (+). PCA^4^ accounted for only 9.30% of the data variation, specifically related to camphor essential oil and camphor content. The PCA analysis identified four main groups (PCA^1^, PCA^2^, PCA^3^, and PCA^4^) based on factor loads (Table 5). Pearson's correlation coefficients were counted to examine the relationships between total phenolic content (TPC), total flavonoid content (TFC), terpenoids, and the antioxidant activity of extracts from yarrow using DPPH and Ferrous ions chelating assays. These coefficients are shown in Table 6. 1,8‐Cineole (%) showed strong positive correlations with terpinen‐4‐ol (%) (0.695**), 1,8‐cineole (μg mL^−1^) (0.724**), camphor (μg mL^−1^) (0.679**), DPPH radical scavenging (0.542**), and Ferrous ions chelating assay (0.499**), but had negative correlations with TFC (−0.530**) and TPC (−0.573**). Camphor (%) was positively correlated with borneol (%) (0.526**), camphor (μg mL^−1^) (0.852**), and borneol (μg mL^−1^) (0.569**), but negatively correlated with cis‐4‐thujanol (%) (−0.385*), 1,8‐cineole (μg mL^−1^) (−0.455**), and TPC (−0.374*). Borneol (%) had strong positive correlations with lavandulol (%) (0.653**) and borneol (μg mL^−1^) (0.856**), but negative correlations with cis‐4‐thujanol (%) (−0.552*), terpinen‐4‐ol (%) (−0.468**), and 1,8‐cineole (μg mL^−1^) (−0.642**). Cis‐4‐thujanol (%) was positively correlated with 1,8‐cineole (μg mL^−1^) (0.680**), DPPH radical scavenging (0.556**), and Ferrous ions chelating assay (0.620**), but negatively correlated with lavandulol (%) (−0.651**), borneol (μg mL^−1^) (−0.610**), and essential oil yield (−0.435*). Lavandulol (%) showed positive correlations with 1,8‐cineole (μg mL^−1^) (0.642**), camphor (μg mL^−1^) (0.852**), and borneol (μg mL^−1^) (0.569**), but negative correlations with terpinen‐4‐ol (%) (−0.608**), Ferrous ions chelating assay (−0.422*), and TPC (−0.520**). Terpinen‐4‐ol (%) was positively correlated with camphor (μg mL^−1^) (0.656**) and Ferrous ions chelating assay (0.492**). 1,8‐Cineole (μg mL^−1^) had strong positive correlations with camphor (μg mL^−1^) (0.727**), borneol (μg mL^−1^) (0.745**), and essential oil yield (0.620**), but negative correlations with TFC (−0.395**) and TPC (−0.356*). Camphor (μg mL^−1^) was negatively correlated with TPC (−0.622**). Essential oil yield was positively correlated with TFC (0.554**). Additionally, the DPPH radical scavenging assay was strongly positively correlated with the Ferrous ions chelating assay (0.993**) and TFC (0.518**). TFC was positively correlated with TPC (0.678**).

**TABLE 5:** Factor loadings obtained by principal components analysis.

Component	PCA_1_	PCA_2_	PCA_3_	PCA_4_
1,8‐Cineole (%)	0.233	0.769	0.011	−0.131
Camphor (%)	0.042	0.100	0.220	0.615
Borneol (%)	0.065	0.116	0.759	−0.225
Cis‐4‐thujanol (%)	0.961	0.130	−0.132	0.093
Lavandulol (%)	0.964	0.030	−0.021	−0.082
Terpinen‐4‐ol (%)	0.782	0.171	0.258	−0.355
1,8‐Cineole(μg mL^−1^)	0.000	0.875	−0.248	0.235
Camphor (μg mL^−1^)	0.103	0.428	−0.032	0.845
Borneol (μg mL^−1^)	0.175	0.314	0.788	0.221
Essential oil yield	0.676	0.126	0.069	0.107
DPPH radical scavenging assay	−0.183	−0.100	0.924	−0.176
Ferrous ions chelating assay	0.058	0.012	0.748	0.042
Total phenolic content	−0.203	0.702	−0.219	−0.354
Total flavonoid content	−0.224	0.768	−0.241	0.347
Eigenvalue	4.62	2.58	1.41	1.36
Explained variance (%)	40.20	26.53	15.45	9.30
Total variance (%)	40.20	66.73	81.18	90.48

Abbreviation: DPPH, 2,2‐diphenyl‐1‐picrylhydrazyl.

**TABLE 6:** Pearson's correlation coefficients between bioactive compounds and antioxidant capacity of *Achillea gypsicola*.

Traits	1,8‐Cineole (%)	Camphor (%)	Borneol (%)	Cis‐4‐thujanol (%)	Lavandulol (%)	Terpinen‐4‐ol (%)	1,8‐Cineole (μg mL^−1^)	Camphor (μg mL^−1^)	Borneol (μg mL^−1^)	EOY	DPPH	FI	TFC	TPC
1,8‐Cineole (%)	1													
Camphor (%)	0.017	1												
Borneol (%)	−0.101	0.526**	1											
Cis‐4‐thujanol (%)	0.189	−0.385*	−0.552**	1										
Lavandulol (%)	−0.003	0.131	0.653**	−0.651**	1									
Terpinen‐4‐ol (%)	0.695**	−0.017	−0.468*	0.385*	−0.608**	1								
1,8‐Cineole (μg mL^−1^)	0.724**	−0.455**	−0.642**	0.680**	0.720*	0.642*	1							
Camphor (μg mL^−1^)	0.679**	0.852**	−0.308	0.180	0.856**	0.656**	0.727**	1						
Borneol (μg mL^−1^)	−0.116	0.569**	0.856**	−0.610**	0.763**	0.474*	0.745**	−0.067	1					
EOY	0.421*	−0.014	0.505**	−0.435*	0.805**	−0.292	0.620**	0.054	0.400*	1				
DPPH	0.542**	0.456*	0.438*	0.556**	−0.317	0.449*	0.415*	0.328	0.437*	0.208	1			
FI	0.499**	0.467*	0.472*	0.620**	−0.422*	0.492**	0.420*	0.327	0.497**	0.089	0.993**	1		
TFC	−0.530**	−0.293	−0.224	−0.199	−0.219	−0.006	−0.395**	−0.369*	−0.301	0.554**	0.518**	−0.372*	1	
TPC	−0.573**	−0.374*	−0.259	−0.280	−0.520**	0.389*	−0.356*	−0.622**	−0.034	−0.122	0.335*	0.340*	0.678**	1

Abbreviations: DPPH, 2,2‐diphenyl‐1‐picrylhydrazyl; EO, Essential oil; EOY, Essential oil yield; FI, Ferrous ions; TFC, Total flavonoid content; TPC, Total phenolic content.

** and * significant at *p* ≤ 0.01 and *p* ≤ 0.05 probability levels, respectively.

Principal component analysis and correlation coefficients indicated that TFC, and to a lesser extent TPC, were primarily dependable for the antioxidant capacity observed in *A. gypsicola* samples. The data revealed that the proportions of compounds such as borneol, camphor, and 1,8‐cineole in the essential oil significantly influenced DPPH activity and Ferrous ions chelating tests. Previous research has shown notable correlations between antioxidant activity and certain terpenoids (Tohidi‐Nejad et al. 2024). For instance, Wang et al. (2017) found that borneol enhances antioxidant capacity and has potential applications in food. Similarly, Li et al. (2024) identified a positive correlation between borneol and antioxidant activity. Like our results, Kanyal et al. (2024) reported in their study that monoterpenes such as borneol, camphor, and 1,8‐cineole may be responsible for antioxidant activity. Additionally, these studies noted that borneol is synthesized from geranyl diphosphate, a precursor of monoterpenes, with camphor also participating in the same biosynthetic pathway (Ma et al. 2022). Consequently, the findings regarding borneol and camphor in our study corroborate this relationship. Hu et al. (2024) reported that borneol has anti‐inflammatory effects, analgesia, and the ability to overcome biological barriers, in addition to its antioxidant capacity. Researchers reported strong positive correlations between antioxidant activity and TPC and TFC in their studies (Farajpour et al. 2024; Ghasemi et al. 2024; Taibi et al. 2024). Studies have shown that exogenously administered salicylic acid plays an important role in regulating gene expression linked to plant growth, defense responses, and biosynthesis of some classes of secondary metabolites. The mitigating effect of SA on plants under drought stress was found to be associated with the improvement of physiological processes by increasing the content of photosynthetic pigments and water retention in plant tissues. Chitosan is a cationic polysaccharide recognized as a potent stimulator of secondary metabolite accumulation in plants. The beneficial effects of chitosan have been identified in increasing plant tolerance to biotic and abiotic stresses and in promoting secondary metabolite production. Cyclic monoterpenes such as borneol, camphor, and 1,8‐cineole are abundant in nature and highly valued for their biological activities, including antioxidant and antimicrobial effects. These terpenes are among the most prevalent in nature and are commonly used as food additives. Recently, they have been increasingly recognized for their biological effects, including antimicrobial, anti‐inflammatory, anti‐proliferative, and antioxidant activities. Nature‐based compounds offer numerous advantages, as their combined antibacterial and antioxidant properties can address various issues, such as antibiotic resistance and the negative impacts of oxidative stress (Akacha et al. 2022; Ben Akacha et al. 2023a, 2023b, 2023c; Ben Hsouna et al. 2023).

## 4 Conclusion

This work is the first to investigate the effect of SA and CTS practices on the TP and TF contents, antioxidant activity, essential oil composition, and monoterpenes in *A. gypsicola*. The findings provide valuable insights into how *A. gypsicola* responds to various SA and CTS treatments, particularly when applied during the early flowering period. This research highlights the potential for enhancing the phytochemical quality of *A. gypsicola*. Additionally, it advances our understanding of the dynamic antioxidant capacity and SMs of this plant. The results suggest that manipulating *A. gypsicola* with SA and CTS can effectively boost SMs accumulation. Foliar applications of SA and CTS are shown to be simple and cost‐effective methods for increasing the accumulation of specific terpenoids in *A. gypsicola*. This approach enhances the quality of yields and shows a giving method for growing MAPs with improved wellness and pharmaceutic profits. Notably, camphor and 1,8‐cineole synthesis increased by 213.2% (8 g L^−1^ CTS) and 125.1% (4 g L^−1^ CTS), respectively. The highest levels of TFC and TPC were observed with CTS 8 g L^−1^ and SA 2 mM treatments, reaching 175.5 mg QE g^−1^ DW and 468.1 mg GAE g^−1^ DW, respectively. The greatest amounts of borneol, camphor, and 1,8‐cineole were achieved with SA 2 mM and CTS 8 g L^−1^ treatments, showing increases of 0.62‐fold (from 37.20 to 60.19 μg mL^−1^), 2.13‐fold (from 63.4 to 198.6 μg mL^−1^), and 1.25‐fold (from 78.1 to 175.8 μg mL^−1^), respectively. Significant improvements were also noted in other essential components. Our results showed that the exogenous application of SA and CTS not only increased the secondary metabolite unit but also contributed to the improvement of antioxidant capacity in *A. gypsicola*. SA and CTS treatment also increased the accumulation of some compounds with proven pharmacological properties in *A. gypsicola*. It is thought that SA and CTS applications may contribute significantly to the accumulation of some secondary compounds in plants when applied at full flowering.

## Author Contributions

**Muhammed Akif Açıkgöz:** conceptualization (equal), visualization (equal), writing – original draft (equal), writing – review and editing (equal). **Ebru Batı Ay:** funding acquisition (equal), investigation (equal). **Beril Kocaman:** data curation.

## Conflicts of Interest

The authors declare no conflicts of interest.

## Data Availability

Data will be made available on request.
